# Influence of a Prolonged Tennis Match Play on Serve Biomechanics

**DOI:** 10.1371/journal.pone.0159979

**Published:** 2016-08-17

**Authors:** Caroline Martin, Benoit Bideau, Paul Delamarche, Richard Kulpa

**Affiliations:** M2S Laboratory, UFR APS, University of Rennes 2 - ENS de Rennes, Campus de Ker Lann, Avenue Robert Schuman, 35170, Bruz, France; Duke University, UNITED STATES

## Abstract

The aim of this study was to quantify kinematic, kinetic and performance changes that occur in the serve throughout a prolonged tennis match play. Serves of eight male advanced tennis players were recorded with a motion capture system before, at mid-match, and after a 3-hour tennis match. Before and after each match, electromyographic data of 8 upper limb muscles obtained during isometric maximal voluntary contraction were compared to determine the presence of muscular fatigue. Vertical ground reaction forces, rating of perceived exertion, ball speed, and ball impact height were measured. Kinematic and upper limb kinetic variables were computed. The results show decrease in mean power frequency values for several upper limb muscles that is an indicator of local muscular fatigue. Decreases in serve ball speed, ball impact height, maximal angular velocities and an increase in rating of perceived exertion were also observed between the beginning and the end of the match. With fatigue, the majority of the upper limb joint kinetics decreases at the end of the match. No change in timing of maximal angular velocities was observed between the beginning and the end of the match. A prolonged tennis match play may induce fatigue in upper limb muscles, which decrease performance and cause changes in serve maximal angular velocities and joint kinetics. The consistency in timing of maximal angular velocities suggests that advanced tennis players are able to maintain the temporal pattern of their serve technique, in spite of the muscular fatigue development.

## Introduction

The typical average tennis match duration is between 1 and 2 hours but in some cases this duration can be prolonged (from 3 to 6 hours) [1] [2]. In Grand Slam Tournaments, the mean duration of 5-set matches is between 137 and 154 minutes, according to the court surface [3]. Tennis match play is defined by intermittent exercise: short bouts of high intensity (< 10 seconds) are interrupted by short active recovery bouts (10–20 seconds) and passive recovery periods of longer duration (90–120 seconds). Throughout an extreme five set tennis match, players can hit more than 1000 groundstrokes and 400 serves [2] leading to muscular fatigue, which is considered both as a cause of performance impairment and an injury risk factor [4].

The influence of muscular fatigue on serve ball speed during tennis is unclear since previous studies have reported conflicting results. Indeed, it has been shown that serve ball speed did not change between the beginning and the end of 2h30 and 4-hours tennis matches [5] [6], while it has been reported that a 2-hour tennis match or training session decreased serve accuracy (from -12 to -30%), ball speed (-4.5%) and increased percentage of errors [7][8][9]. Moreover, the effects of tennis fatigue on muscle capacities have been previously analyzed [8][10]. Electromyographic activity (EMG) and force measured during isometric maximal voluntary contraction decreased in knee extensors, in plantar flexors during a 3-hour tennis match [10], in pectoralis major and flexor carpi radialis during a 40-min tennis exercise composed by 4 series of 12 repetitions of 1 serve followed by 8 forehand strokes [8]. However, this last result was measured during “artificial” fatigue protocol that may fail to reflect fatigue level obtained for prolonged tennis match [4]. Indeed, one of the limitations in these previous studies [7][8][9] is that the duration of tennis exercise ranged from 40 to 120 minutes, and the total number of serves was limited to 100 or fewer. Yet, it is essential that scientific researches reflect the true competition situation to accurately understand the fatigue effects on tennis performance and muscular activity [4].

Previous studies carried out in other activities have shown that muscular fatigue can decrease ball speed (from -2.9 to -5.6%), alter kinetics (from -4.8 to -15.2%) and kinematic (from -3.8 to -14%) in baseball throwing [11], [12] and in football kicking [13]. To our knowledge, no study has analyzed the influenced of upper limb muscular fatigue on serve biomechanics during a 3-hour tennis match. Yet, understanding the ways by which muscular fatigue can influence serve biomechanics and performance has considerable interests for tennis players, coaches and medical practitioners.

Because altered serve biomechanics due to muscular fatigue may be detrimental to a tennis player performance and increase injury risk, this study aims to quantify kinematic, kinetic and performance changes that occur in the tennis serve throughout a prolonged match. It is expected that muscular fatigue induced by a 3-h tennis match can lead to significant decreases in maximal angular velocities, ball speed and impact height and changes in joint kinetics.

## Materials and Methods

### Participants

Eight right-handed male tennis players (mean ± SD: age 20.4 ± 2.8 years; height 1.80 ± 0.05 m; weight 69.4 ± 9.8 kg; weekly tennis training: 5.8 ± 2.6 hours, weekly conditioning training: 1.3 ± 0.5 hours, tennis experience: 12.2 ± 2.9 years), with an International Tennis Number between 3 and 4 have participated voluntarily in this study. Prior to experimentation, the participants were fully informed of the experimental procedures. Written consent was obtained for each player. The study was approved by the Research Ethical Committee of the M2S Laboratory from the University of Rennes 2 and conducted in accordance with the 1975 Declaration of Helsinki.

### Experimental protocol

**Fig 1:**
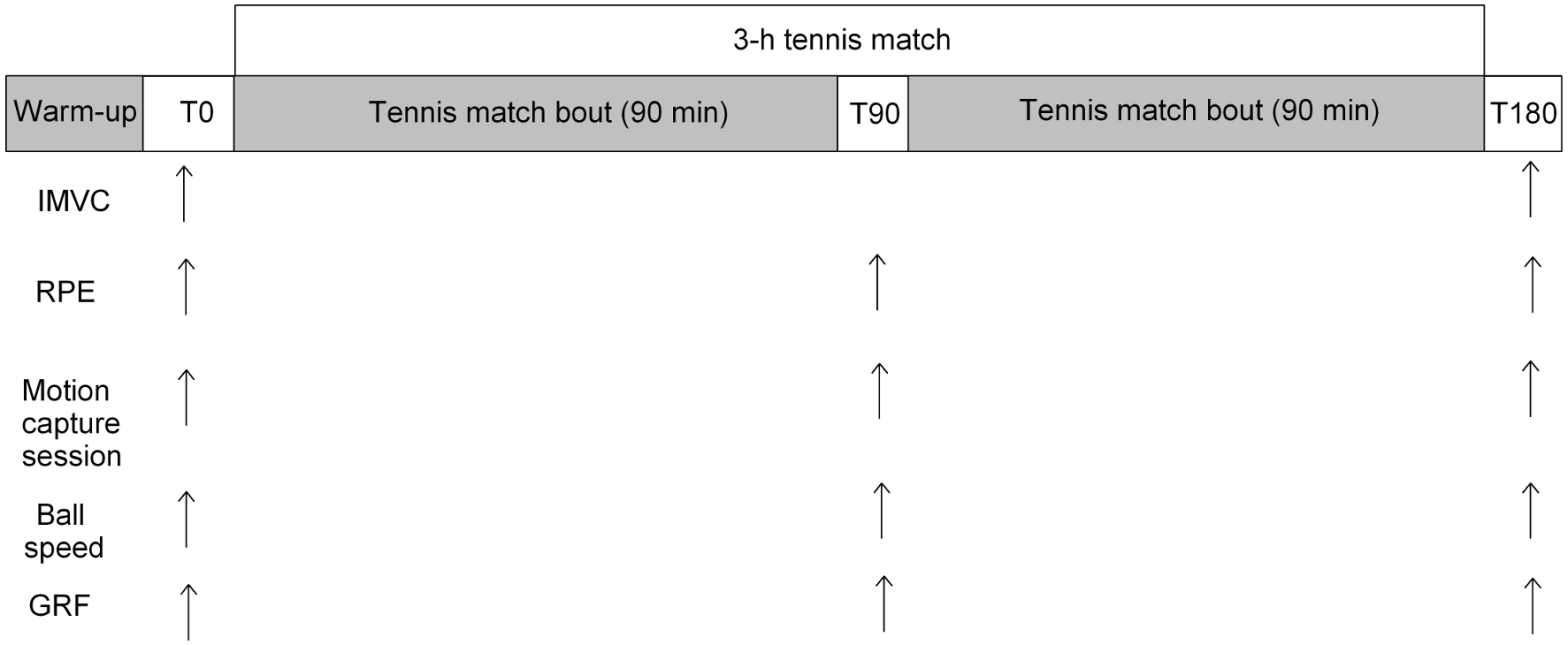
Experimental protocol. IMVC: isometric maximal voluntary contraction, RPE: rating of perceived exertion, Sball: ball speed, GRF: ground reaction forces.

The prolonged exercise was a three-hour competitive tennis match (Fig 1). All subjects played against an opponent of similar standard on an indoor tennis court. Each tennis match was preceded by a standard warm-up, as practiced during tennis tournaments. With the exception of the serve motion capture session at mid-match (T90), the matches were played according to the rules of the International Tennis Federation. The resting times allowed were 20 seconds between points, 90 seconds between change-overs, and 120 seconds between sets. Subjects were asked to play at their best level as in an official tournament. The experimental protocol was designed to measure EMG data during isometric maximal voluntary contraction, serve kinetic and kinematic values during motion capture sessions, RPE, ball speed, vertical ground reaction forces (GRF).

#### Isometric maximal voluntary contraction (IMVC)

Before (T0) and after each match (T180), surface electrodes (Comepa, electrode diameter: 1 cm, inter-electrode distance: 2 cm) were placed over the following eight muscles of the dominant limb: anterior deltoid, middle trapezius, pectoralis major, serratus anterior, latissimus dorsi, biceps brachii, infraspinatus, triceps brachii. The electrodes were placed in the middle of muscle bulks and aligned in parallel with the muscle fibres based on the recommendations of the surface electromyography (EMG) for the non-invasive assessment of muscles (SENIAM) Project [14]. To avoid EMG limitations, electrode locations were determined using standard procedures for each muscle [15] [16]. Before placing the surface electrodes, skin surface was shaved and cleaned with alcohol to limit the potential artefact-imposing effects of sweat. The wires connected to the electrodes were maintained with tape to avoid noise from upper limb movements. The electrodes were taken off at the end of the first EMG data registration (T0) and were replaced after the match (T180). To make sure that the electrodes’ placement was similar between T0 and T180, electrodes positions at T0 were circled with a felt-tip skin marker. Electrode placements were confirmed by analysing EMG signal to noise ratio during IMVC tests.

EMG signals were recorded with the Wave Wireless EMG system (Cometa, Italy, 2000 Hz). The total system amplification gain was set at 1000. Once the electrodes were placed, IMVC were performed for each muscle using standard manual muscle testing positions which were strictly standardized [17]. For all IMVC tests, participants were strictly controlled and instructed to maintain the required position. Each IMVC test lasted 5 seconds with a rapid increase of contraction over 1 second, a sustained maximum for 3 seconds, and a progressive release during the last second. Players were strongly encouraged to perform IMVC at their maximum level. The EMG signals of IMVC were filtered using a Butterworth filter with band-pass of 20–500Hz and then the data were full-wave rectified. A fast Fourier transform was used to compute the mean power frequency (MPF) of the power spectrum for analysis of muscle fatigue. The definition of MPF is given by

MPF= ∑j=1MfjPj∑j=1MPj

where *f*_*j*_ is the frequency value of EMG power spectrum at the frequency *j*, *P*_*j*_ is the EMG power spectrum at the frequency *j*, and *M* is the length of frequency *j*. Indeed, mean power frequency is used as indicator of muscle fatigue [18]. A minimum reduction of 8% in the MPF value was considered to be an indication of local muscle fatigue [19].

#### Serves motion capture session

The motion capture sessions of serves were performed before (T0), at mid-match (T90) and immediately after (T180) a 3-hour match. For each session, the players performed 5 successful ‘flat’ serves from the right service court to a 1.50 x 1.50 m target area bordering the T of the “deuce” service box. The numbers of serve trials for each player have been recorded. Thirty-eight retro-reflective markers were placed on the following anatomical landmarks: sterno-clavicular joint, xiphoid process, 7th cervical vertebra, 10th thoracic vertebra, and for both hemi-bodies, occipital and frontal bones, glenohumeral joint, lateral humeral epicondyle, radius head, head of the third metacarpus, ulnar styloid process, radial styloid process, anterior superior iliac spine, posterior superior iliac spine, fibula lateral condyle, tibia medial condyle, lateral and medial malleolus, heel, acropodion and head of the second metatarsus. These anatomical landmarks were determined in agreement with previously published data [20] [21] [22]. Five landmarks were positioned on the racket: mid-height of both racket-face sides, bottom of the handle, top and bottom of the racket-face [23]. Three additional landmarks were positioned on the ball to compute ball impact height, as described in previous study [24]. Players wore only tight shorts to limit movement of the markers from their anatomical landmarks. The landmarks were taken off at the end of the first EMG data registration (T0) and were replaced after the match (T180). To make sure that the landmarks’ placement was similar between T0 and T180, landmarks positions at T0 were circled with a felt-tip skin marker.

A Vicon MX-40 motion capture system (Oxford Metrics Inc., Oxford, UK) was used to record the 3D landmarks trajectories. It was composed of 12 high-resolution cameras (4 megapixels) operating at a nominal frame rate of 200 Hz. The error of the motion capture system is less than 1mm. After the capture, 3D coordinates of the landmarks were reconstructed with ViconIQ software (IQ, Vicon, Oxford, UK) with a residual error less than 1 mm. The 3D motions of each player were expressed in a right-handed inertial reference frame, where the origin was at the center of the baseline. X was along the baseline, Y pointed forward and Z was vertical and pointed upward. The 3D coordinate data of the markers were smoothed with a Butterworth low-pass filter with a cutoff frequency of 10 Hz, determined by residual analysis [25].

#### Kinematic values

Flexion angle of the back knee was determined as the external relative angle between the thigh and the shank (full knee extension equals 0° of flexion) [26]. The maximal angular velocity values of back knee extension, trunk transverse rotation (also called trunk somersault rotation by tennis coaches and experts in biomechanics [27]), trunk sagittal rotation (also called shoulder-over-shoulder rotation by tennis coaches and experts in biomechanics [27]), pelvis and upper torso longitudinal rotations, elbow extension, wrist flexion and shoulder internal rotation have been calculated from the serves monitored during motion capture sessions. Angular velocities were computed in the inertial reference frame by using the following equation:

$${\left[\omega \right]}_{I}=\;[\dot{R}]{\left[R\right]}^{T}$$

Where $\left[\dot{R}\right]$ is the derivate of the rotation matrix from the local orthogonal reference frames (attached to the body joints) to the inertial reference frame, [*R*]^*T*^ is the transpose of the same rotation matrix. Moreover, the times of these maximal angular velocities have been measured and expressed as a function of the normalized serve duration, defined from ball toss (0%) to ball impact (100%). Ball toss and ball impact were determined by direct observation of the recorded data. These parameters were selected because they delimit a sequence of body motions during which the transfer of energy and momentum from the lower limbs to the upper limb occurs when performing tennis serve [28] [29]. All the kinetic and kinematic values were calculated by Matlab software 6.5 (Mathworks, Natick, Massachussetts, USA).

#### Maximal values of upper limb joint kinetics

Maximal kinetic values of the shoulder, elbow and wrist joints were calculated. These kinetic values have been chosen because they are thought to be indicative of injury potential during tennis serve movements [26] [23]. The serving arm was modeled as a 3-link kinetic chain composed of the racket/hand segment, forearm and upper arm. The inverse dynamic with a top-down approach was used to calculate the joint forces and torques. The joint forces and torques obtained for the shoulder and elbow joints were first computed in terms of inertial reference frame and were later transformed to a series of non-inertial, anatomically relevant, right-handed orthogonal reference frames at each joint, by using the procedure described by Feltner and Dapena (1986) [30]. Moment of inertia of the racket about its medio-lateral axis was computed using the parallel axis theorem and published racket “swingweight” data [31]. Racket moment of inertia about the long-axis was calculated as reported in the literature [32]:

$$moment\;of\;inertia\;\left(kg\cdot m^{-2}\right)=\left(mass\;\times \;head\;width^{2}\right)/17.75$$

Racket moment of inertia about its antero-posterior axis was the sum of the racket’s other two principal moments of inertia [32]. Segmental masses and moments of inertia used in the inverse dynamics calculations were obtained from previously published data [33].

#### Ground reaction forces (GRF)

A force platform (60 x 120 x 5.7 cm, Advanced Mechanical Technology Incorporation, Watertown, MA, USA) was used to measure peak of vertical ground reaction forces during serve motion capture sessions. For each trial, peak of vertical GRF was identified using Matlab software (3.7.2, Biopac, Santa Barbara, CA). 6.5 (Mathworks, Natick, Massachussetts, USA).

#### Serve performance parameters

For each serve performed during the motion capture sessions, ball impact height was measured. Post-impact ball speed was measured for each trial with a radar (Stalker Professional Sports Radar, Plano, TX, precision: ± 1.6 km/h, frequency: 34.7 GHz, Target Acquisition Time: 0.01 sec) fixed on a 2.5 m height tripod, 2 m behind the players in the direction of the serve. Retro—reflective scotch bouts were stuck to the ball in order to capture ball toss and to measure impact height.

#### Rating of Perceived Exertion

The subjective score of rating of perceived exertion (RPE) from 6 to 20 [34] was measured every 90 minutes during the match. In tennis, RPE score is a common indicator often used to quantify the subjective perceived exertion [35][36].

#### Match characteristics

Each match was video recorded to determine the score, the total number of groundstrokes and serves hit by the players, mean duration of rallies, effective playing time and number of strokes per point. The effective playing time was calculated by dividing the sum of the single rally duration by the total match duration, as previously reported in the literature [37]. The effective playing time was expressed as the percentage of the total match duration. The mean duration of rallies was determined by dividing the sum of rally duration by the number of rallies played during the match [37].

### Statistical analyses

Means and standard deviations (see S1 file for raw data) were calculated for all variables. The data distribution was not normal. Consequently, data (ball speed, RPE, GRF, kinetic and kinematic values) were tested with Friedman’s repeated measures analysis of variance on ranks. When significant main effects were found, the Student Newman Keuls Method post-hoc test was used. Paired T-tests were used to compare MPF values between T0 and T180. When the normality test failed, a Wilcoxon signed ranks test was used. Effect size (ES) was calculated to document the size of the statistical effects observed and defined as small for ES > 0.1, medium for ES > 0.3 and large for ES > 0.5 [38]. Statistical significance was accepted at P < 0.05. The experiment-wise type I error rate was not controlled. The statistical analyses were undertaken by using SigmaStat software (Jandel Corporation, San Rafael, CA, USA).

## Results

During the tennis matches, the participants played 5 sets and 46 ± 1 games. Moreover, they hit 547 ± 93 groundstrokes and 253 ± 25 serves. Mean duration of rallies, effective playing time and number of strokes per point were 7.4 ± 0.8 s, 21.8 ± 2.0%, and 5.0 ± 0.5 strokes respectively. The number of serve trials was not significantly different for the capture sessions between T0 (10.4 ± 2.4 trials), T90 (10.3 ± 3.2 trials) and T180 (10.1 ± 2.2 trials) (p = 0.556).

### MPF values and RPE

Biceps brachii, anterior deltoid, pectoralis major, middle trapezius, and triceps brachii showed significant decreases of MPF when comparing IMVC tests performed at T0 and T180 (Table 1). Cohen’s effect size values suggested a large significance (ES > 0.5). There was a trend toward a MPF decrease in infraspinatus from T0 to T180 (p = 0.096). There was no significant MPF difference in serratus anterior and latissiumus dorsi before and after the 3-hour tennis match (Table 1). The RPE score increased significantly during the 3-hour tennis match from T0 to T180 (T0: 7.2 ± 1.6; T90: 12.5 ± 1.9; T180: 17.3 ± 1.3) (p<0.001) (ES = 0.72).

**Table 1:** Mean power frequency of the muscles tested during IMVC before (T0) and immediately after the match (T180). Values are mean ± SD.

Muscles	MPF at T0 (Hz)	MPF at T180 (Hz)	P	Change (%)	Effect size (ES)
Biceps brachii	132.8 ± 8.8	121.1 ± 15.8	0.039	-8.8	0.525
Anterior deltoid	156.5 ± 51.6	135.7 ± 12.0	0.023	-13.3	0.560
Serratus anterior	135.6 ± 17.1	132.7 ± 9.6	0.438	/	/
Latissimus dorsi	131.6 ± 13.4	130.3 ± 15.8	0.702	/	/
Pectoralis major	136.7 ± 18.5	124.0 ±15.7	0.001	-9.3	0.800
Infraspinatus	161.1 ± 24.1	150.8 ± 17.2	0.096	-8.4	0.588
Middle trapezius	136.0 ± 15.9	131.9 ± 16.2	0.009	-3.0	0.804
Triceps brachii	168.3 ± 27.1	152.4 ± 31.7	0.029	-9.5	0.720

### Serve performance parameters

The results show that all serve performance parameters were significantly different between T0 and T180. Ball speed at the end of the match (T180: 43.8 ± 4.1 m.s^-1^) was significantly reduced in comparison with T0 (45.6 ± 3.1 m.s^-1^, p = 0.002) (ES = 0.46) and T90 (44.6 ± 3.4 m.s^-1^, p = 0.002) (ES = 0.33). Ball impact height before the match (T0: 2.65 ± 0.08 m) was significantly higher than at T90 (2.63 ± 0.08 m, p = 0.005) (ES = 0.34) and T180 (2.61 ± 0.06 m, p<0.001) (ES = 0.41).

### Lower body serve biomechanics

Results show that two lower body serve variables (maximal back knee flexion angle and maximal back knee extension angular velocity) were significantly different across time while no statistically significant difference was observed for the peak of vertical GRF during the match (p = 0.839). Maximal back knee flexion angle at T0 was significantly higher than at T90 (ES = 0.62) and at T180 (p<0.001) (ES = 0.44). Maximal rear knee extension angular velocity was significantly decreased during the match (p<0.001) (ES = 0.70) (Table 2).

**Table 2:** Lower limb serve biomechanics before (T0), at mid-match (T90) and immediately after the match (T180). Values are mean ± SD.

	T0	T90	T180
Maximal knee flexion angle (°)	80 ± 14	75 ± 15***	75 ± 15***
Maximal rear knee extension angular velocity (°/s)	536 ± 142	488 ± 161***	466 ± 164*** $

***p < 0.001; significantly different from T0;

^$^: p < 0.001; significantly different from T90.

### Upper body serve biomechanics

**Fig 2:**
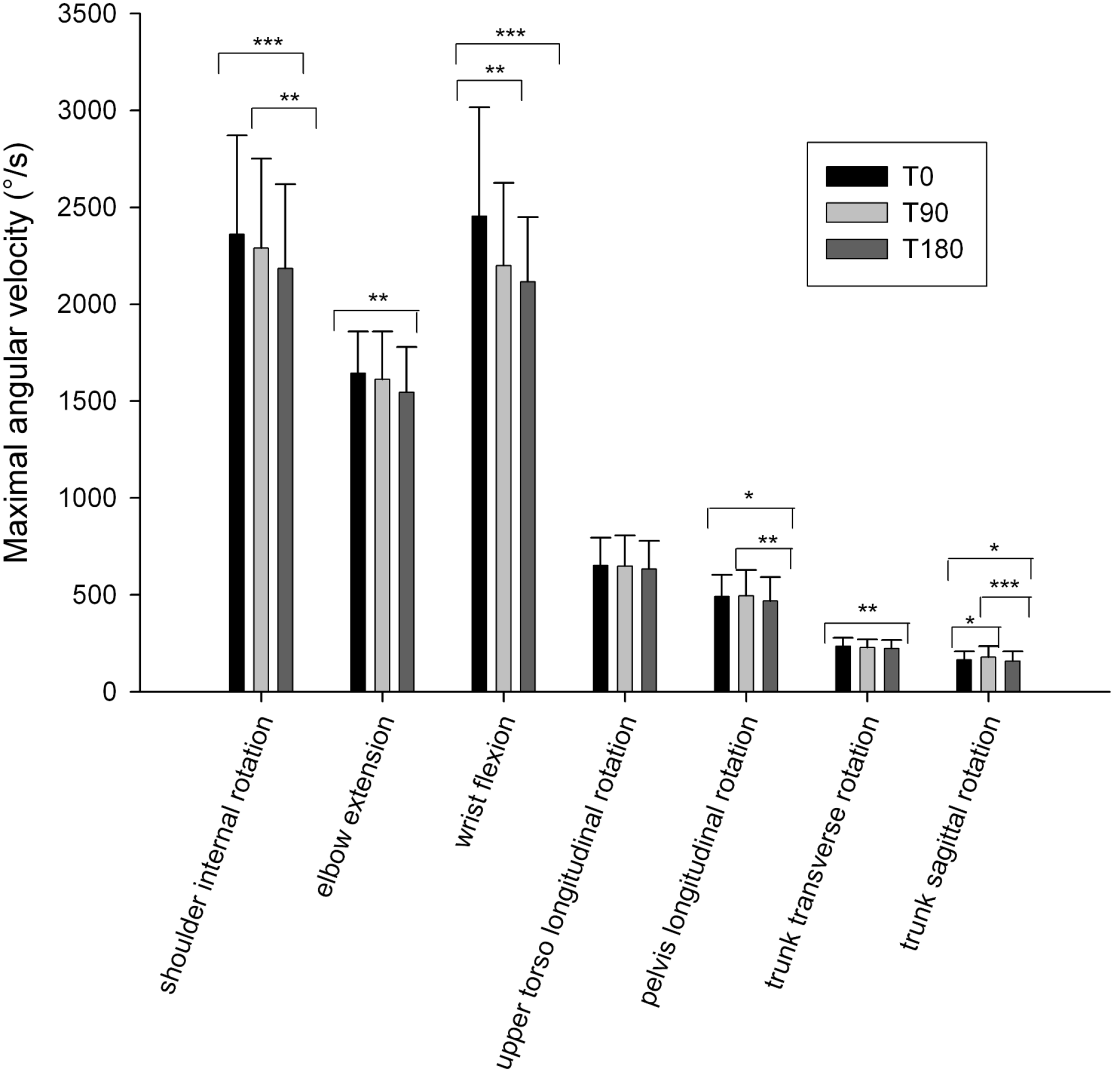
Upper body maximal angular velocities before (T0), at mid-match (T90) and immediately after the match (T180). Values are mean ± SD. ***p < 0.001; ** p <0.01; * p < 0.05.

Results reveal that 16 upper body serve variables were significantly different between T0 and T180 whereas 12 upper body serve variables were unchanged. Between T0 and T180, the results show significant decreases in maximal angular velocity of shoulder internal rotation (-7.5%) (p = 0.003) (ES = 0.46), elbow extension (-6.0%) (p<0.001) (ES = 0.57), wrist flexion (-13.8%) (p<0.001) (ES = 0.61), pelvis longitudinal rotation (-4.7%) (p = 0.021) (ES = 0.36), trunk transversal rotation (-5.1%) (p = 0.002) (ES = 0.46), and trunk sagittal rotation (-6.0%) (p = 0.034) (ES = 0.33) (Fig 2).

Conversely, no statistically significant difference was observed for the longitudinal upper torso rotation during the match (p = 0.126). Concerning the timing of maximal angular velocities, no statistically significant difference was observed between T0, T90 and T180 (p>0.05).

**Fig 3:**
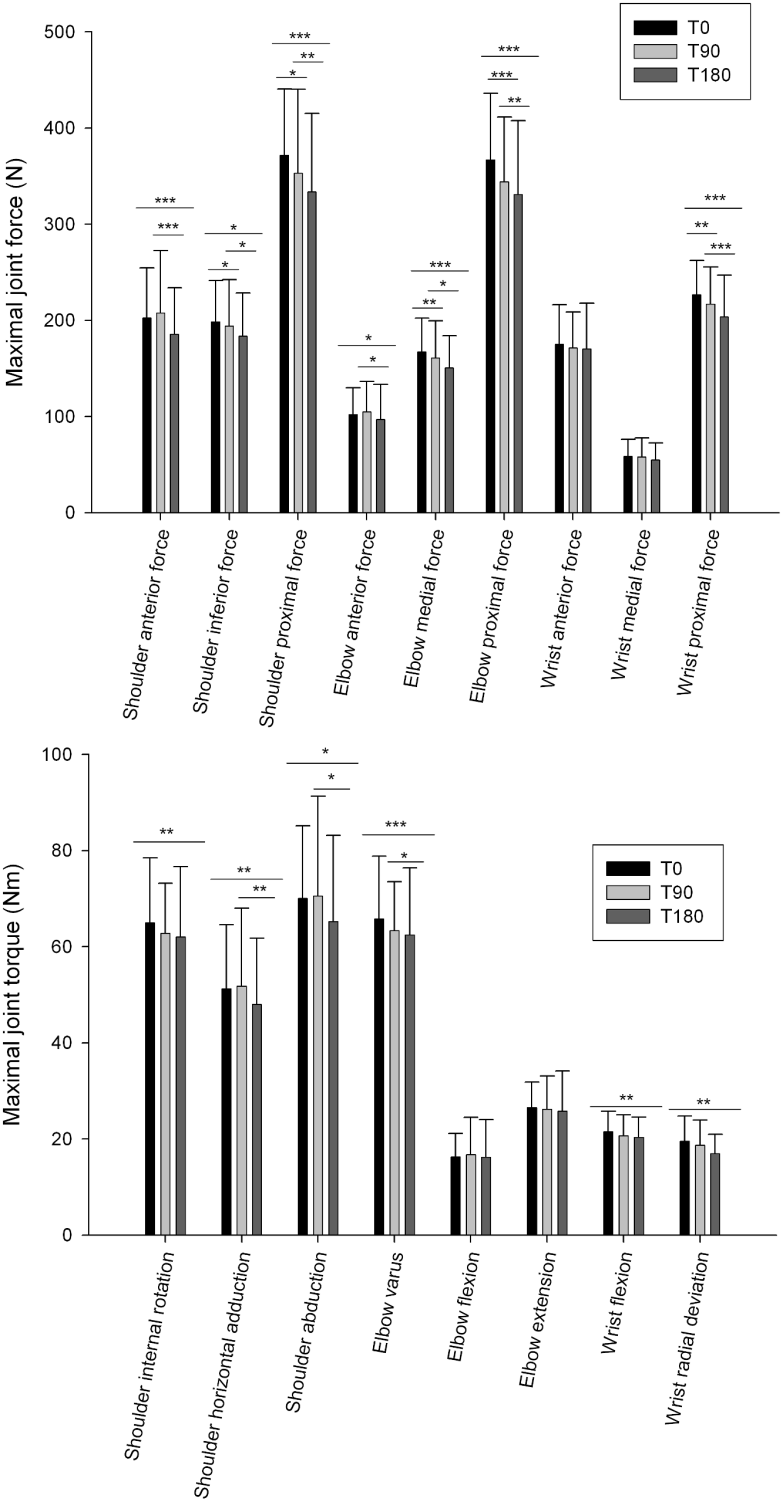
Maximal values of upper limb joint forces and torques before (T0), at mid-match (T90) and immediately after the match (T180). Values are mean ± SD. ***p < 0.001; ** p <0.01; * p < 0.05.

Maximal values of shoulder anterior force (p<0.001) (ES = 0.70), shoulder inferior force (p = 0.024) (ES = 0.35), shoulder proximal force (p<0.001) (ES = 0.66), shoulder internal rotation torque (p = 0.01) (ES = 0.46), shoulder horizontal adduction torque (p<0.001) (ES = 0.42), shoulder abduction torque (p = 0.011) (ES = 0.39), elbow anterior force (p = 0.023) (ES = 0.23), elbow medial force (p<0.001) (ES = 0.73), elbow proximal force (p<0.001) (ES = 0.83) and wrist proximal force (p<0.001) (ES = 0.61) significantly decreased from T0 to T180. However, maximal values of wrist anterior (p = 0.111) and medial forces (p = 0.241), and elbow flexion (p = 0.509) and extension torques (p = 0.248) did not change between T0 and T180 (Fig 3).

## Discussion

This study was the first that quantifies kinematic, kinetic and performance changes occurring during tennis serve throughout a prolonged match. Results show that a 3-hour tennis match significantly decreased serve ball speed, ball impact height, maximal knee and upper limb angular velocities, decreased or maintained upper limb joint kinetics, and increased RPE in advanced male tennis players. Surprisingly, there were no significant differences in timings of maximal angular velocities between T0 and T180. Based on our criteria of an 8% reduction in MPF as a sign of local muscle fatigue, all muscles demonstrated signs of fatigue with the exception of the serratus anterior, latissimus dorsi and middle trapezius muscles.

The mean numbers of serves, groundstrokes, sets and games performed by the players during our matches are largely higher than those reported for 90-minute tennis match during which players hit approximately between 50 and 150 serves, 100 and 300 groundstrokes and performed 2 or 3 sets. RPE score progressively increased between T0 and T180, when players perceived the fatigue as “very high”. RPE values at T180 are in accordance with previous studies about tennis fatigue [35] and testify to the strenuous physical effort sustained by the players during the matches.

Furthermore, results about IMVC show decreases in MPF values (>8%) between T0 and T180 for biceps brachii, anterior deltoid, pectoralis major, infraspinatus and triceps brachii, revealing a shift toward lower frequencies of surface myoelectric signal power spectrum that is an indicator of local muscular fatigue [39]. This local muscular fatigue is hypothesized to increase the risk of shoulder and elbow injuries by altering normal muscle functions during the tennis serve. The biceps brachii contraction improves shoulder anterior stability and allows decreasing the load applied on inferior glenohumeral ligament during arm abduction and external rotation in overhead movements [40]. During the follow-through phase of the serve, the biceps brachii resists shoulder distraction, decelerates and stabilizes elbow joint [41] [42]. In case of muscular fatigue, one may hypothesize that biceps brachii becomes less efficient, that would favor the development of shoulder and elbow instability, one serve after another throughout a prolonged match. Infraspinatus is involved in arm external rotation and abduction, and in glenohumeral stability. A decrease of the infraspinatus activity caused by muscular fatigue could lead to superior and anterior translation of the humeral head during the tennis serve, commonly associated with rotator cuff injuries [8] [42]. The significant decrease in MPF value of middle trapezius is low (-3%). It has been shown that fatigue of shoulder muscles (trapezius, deltoid, serratus anterior, infraspinatus) can induce changes in scapular kinematics during overhead activities and could lead to shoulder injuries [43]. However, further studies are necessary to confirm that upper limb muscular fatigue during a prolonged tennis match disrupts scapular motions.

The 1.8 m.s^-1^ decrease in serve ball speed is in line with previous results [7]. This result could be explained by the decreases in maximal angular velocities measured between T0 and T180: -7.5% for shoulder internal rotation, -6.0% for elbow extension, -13.8% for wrist flexion, -4.7% for pelvis longitudinal rotation, -5.1% and -6.0% for transverse and sagittal trunk rotations. Indeed, all these rotations contribute to increase serve ball speed [44]. Our results highlight the existence of fatigue in muscles responsible for the decrease in shoulder internal rotation (pectoralis major) and elbow extension angular velocities (triceps brachii). The decrease in maximal wrist flexion angular velocity between T0 and T180 could be caused by muscular fatigue in flexor carpi radialis, as shown for the tennis serve during a fatigue protocol [8]. Finally, one may hypothesize that the decreases in trunk maximal angular velocities are the results of fatigue in abdominal muscles (rectus abdominis and obliques) and in erector spinae, which contribute to trunk flexion/extension, lateral tilt and longitudinal rotation during the serve [45]. However, no EMG data about trunk muscles were collected in our study.

Ball impact height significantly decreased from T0 to T180. Ball impact height is a crucial factor affecting tennis serve performance, since the higher the impact, the greater the margin for error at the net [27]. Indeed, according to Brody (2006), a small extra height above the ground for the ball impact location not only results in higher ball speeds, but also increases the window of acceptance for the serve, that is the chance, that it will go in [46]. Decreases in ball impact height and velocity are probably caused by kinematical changes in lower limbs between T0 and T180 such as decrease in maximal rear knee angular velocity and in maximal back knee flexion angle. Indeed, it has been reported that both ball speed and impact height significantly increase with an efficient leg drive: effective back knee flexion and then vigorous knee extension [47]. Although we did not analyze the development of muscular fatigue in lower limbs, previous works can explain our results. Indeed, decreases in maximal isometric voluntary contraction of the right knee extensor muscles and in leg stiffness were measured after a 3-hour tennis match [35].

It is interesting to observe the absence of change in timing of maximal angular velocities suggesting a consistent segmental coordination pattern, as previously reported in tennis players [8]. According to Rota et al. (2014), it seems that fatigue in tennis players preferentially induced an adaptation in muscle activity level rather than changes in the modular organization of the muscle coordination. However, this temporal stability in segmental coordination pattern could be questioned according to the expertise level of tennis players. In this study, it is possible that the players’ level allowed them to use a robust serve technique, in spite of the development of muscular fatigue. But, one may assume that the segmental coordination pattern could be modified in beginners with fatigue development during a prolonged tennis match. Indeed, Aune et al. (2008) have compared the effect of fatigue on motor coordination at different skill levels in table tennis [48]. They showed that expertise enhances potential to adjust motor coordination strategies as a reaction to limit negative effects of physical fatigue. However, further studies have to analyze the stability of segmental coordination pattern at different skill levels in tennis.

Several maximal joint kinetic values were unchanged while the others significantly decreased between T0 and T180. These results are in line with those of Murray et al. (2001) and Apriantono et al. (2006) who reported decreases in shoulder, elbow, and knee kinetics during muscular fatigue protocols, respectively for baseball throwing and football kicking [12] [13]. As suggested by Murray et al. (2001), it is unclear whether the significant changes in joint mechanics were a direct result of fatigue that occurs with extended play or if the body adopted protective mechanisms to minimize the risk of injury over the course of a match or the result of these two phenomena [12]. Finally, it is interesting to notice that all the shoulder kinetics significantly decreased between T0 and T180, while it is only the case for 4 of the 6 kinetic values measured at the elbow and for 3 of the 5 kinetic values at the wrist. These results suggest that compensatory mechanisms at various levels of the coordination kinematic chain may act to delay the effects of fatigue and try to maintain an efficient level of play [5]. Players seem to protect their shoulder as a priority by decreasing kinetics on it, since the shoulder is the most vulnerable and loaded joint during the serve [26]. Conversely, several kinetic values of the most distal joints (wrist and elbow) did not change between T0 and T180, maybe to allow the players to maintain a satisfactory ball speed.

Our study has some limitations. First, our findings describe changes that occurred immediately after the match. Exactly when these patterns changes are appearing during the match and how long they last must be specifically analyzed and will be the topic of future research. EMG signals were only collected for 8 upper limb muscles. The fatigue development on core and lower limb muscles was not measured and the central fatigue was not evaluated. Moreover, authors also note the difficulty of isolation of infraspinatus from cross-talk from other muscles with the use of EMG surface electrodes. Another element is the sample size that is somewhat small because we only included advanced tennis players and their participation was voluntary. Making analyses on advanced players generally leads to small sample size because of the difficulty to recruit them. We probably lacked enough power for any meaningful statistical analysis. Statistical analyses on such population have thus some risk of chance detection. We made numerous statistical comparisons that tend to increase the type I error rate. Consequently, the currents results should be considered as preliminary given the unknown effects that are consistent with a low rate of type I errors in the study. In addition, markers placed on the skin of the players could sligthly increase errors in the kinematic and kinetic calculations despite efforts to minimize them, such as by placing markers on bony prominences with the least amount of skin motion. Gordon and Dapena (2006) [49] reported that upper arm kinematic measurements were unreliable owing to movement artifacts just prior to ball impact during a tennis serve. However, because all the kinematic and kinetic values (excepted shoulder internal rotation angular velocity) in this study were not extracted at or near ball impact, the values estimated were reported with an acceptable degree of error. Finally, the relationship between muscular fatigue observed during IMVC and changes observed on the serve mechanics should be interpreted with care because all the muscles tested in this study are not isometrically contracted during the tennis serve motion. Further studies should provide evidence for this potential relationship.

## Conclusions

In conclusion, a 3-hour tennis match induces decrease in mean power frequency (MPF) values for several upper limb muscles that is an indicator of local muscular fatigue. Moreover, the results show decreases in serve ball speed, ball impact height, maximal angular velocities and an increase in rating of perceived exertion (RPE) score throughout the prolonged tennis match. With fatigue, the majority of the upper limb joint kinetics decreased between T0 and T180. Conversely, no change in timing of maximal angular velocities was observed between T0 and T180. This consistency suggests that advanced tennis players are able to use a robust segmental coordination, which allow them to maintain the temporal pattern of their serve technique, in spite of the muscular fatigue development. Further studies are necessary to test the stability of this segmental coordination pattern at different skill levels during tennis fatigue protocols.

## Supporting Information

S1 File Raw data. (XLSX)
